# MetOrigin 2.0: Advancing the discovery of microbial metabolites and their origins

**DOI:** 10.1002/imt2.246

**Published:** 2024-11-06

**Authors:** Gang Yu, Cuifang Xu, Xiaoyan Wang, Feng Ju, Junfen Fu, Yan Ni

**Affiliations:** ^1^ Children's Hospital Zhejiang University School of Medicine, National Clinical Research Center for Child Health Hangzhou China; ^2^ Key Laboratory of Coastal Environment and Resources of Zhejiang Province, School of Engineering Westlake University Hangzhou China

**Keywords:** mediation analysis, metabolome, metabolic reaction, microbiome, origin analysis, orthology analysis, phenotype

## Abstract

First introduced in 2021, MetOrigin has quickly established itself as a powerful web server to distinguish microbial metabolites and identify the bacteria responsible for specific metabolic processes. Building on the growing understanding of the interplay between the microbiome and metabolome, and in response to user feedback, MetOrigin has undergone a significant upgrade to version 2.0. This enhanced version incorporates three new modules: (1) Quick search module that facilitates the rapid identification of bacteria associated with a particular metabolite; (2) Orthology analysis module that links metabolic enzyme genes with their corresponding bacteria; (3) Mediation analysis module that investigates potential causal relationships among bacteria, metabolites, and phenotypes, highlighting the mediating role of metabolites. Additionally, the backend MetOrigin database has been updated with the latest data from seven public databases (KEGG, HMDB, BIGG, ChEBI, FoodDB, Drugbank, and T3DB), with expanded coverage of 210,732 metabolites, each linked to its source organism. MetOrigin 2.0 is freely accessible at http://metorigin.met-bioinformatics.cn.

## Highlights

- MetOrigin 2.0 has integrated three analytical modes and eight distinct modules for advanced microbial metabolite discrimination.
- The updated database and three new features including quick search, orthology analysis, and mediation analysis expand the platform's versatility and functionality.
- MetOrigin 2.0 serves as a comprehensive web server for microbiome‐metabolome data integration, streamlining the identification of microbial metabolites and their origins.

## Graphical abstract

MetOrigin serves as a powerful web‐based tool for microbiome‐metabolome data integration, helping researchers pinpoint microbial metabolites and trace their origins. The newly upgraded MetOrigin 2.0 comes equipped with three analytical modes and eight enhanced/new modules for more precise microbial metabolite identification, including quick database search, origin analysis, pathway analysis, Sankey network, correlation analysis, orthology analysis, mediation analysis, and network visualization. The backend database has been updated with the latest data from seven key public databases (KEGG, HMDB, BIGG, ChEBI, FoodDB, Drugbank, and T3DB), with expanded coverage of 210,732 metabolites, each linked to its source organism.

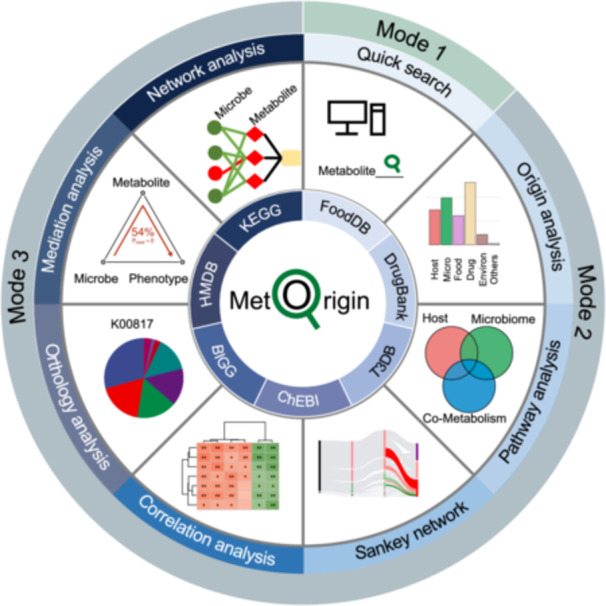

## INTRODUCTION

MetOrigin is a user‐friendly web‐based platform dedicated to the identification of microbial metabolites and the integration analysis of microbiome and metabolome [1]. Since its introduction, it has rapidly gained wide attention within the metagenomics and metabolomics communities. To date, it processes an average of 4000 jobs per month, with a user base exceeding 1800 researchers from diverse research fields, including clinical and public health, drug discovery, nutrition science, food and agriculture, and animal science [2, 3, 4].

Microbial metabolites serve as crucial intermediates and signaling molecules in host‐microbiota interactions, offering promising strategies for preventing and/or treating metabolic diseases [5, 6]. In fact, small bioactive compounds produced by microorganisms form the foundation of numerous therapeutic drugs [7]. Recent research studies have discovered novel microbial bile acids, such as 3‐succinylated cholic acid (3‐sucCA) and 3‐oxolithocholic acid (3‐oxoLCA), produced by specific gut microbiota (GM) species [8, 9]. These compounds exhibit significant clinical and translational potential in alleviating metabolic diseases and inflammatory disorders. Although direct experimental verification is the most accurate strategy, it is challenging and labor‐intensive to conduct the screening of bacterial isolates responsible for producing specific metabolites. Researchers may need to screen hundreds of culturable bacterial colonies, incubate them with chemical substrates under various conditions, and perform quantitative analysis of metabolites to verify their metabolic processes [8, 9]. To address these challenges, advanced bioinformatic tools and comprehensive databases have been developed to efficiently predict microbial metabolites using either single‐omics data or integrative multi‐omics approaches, such as genomics, transcriptomics, proteomics, and metabolomics [10]. Leveraging structural and functional information, prediction tools like antiSMASH [11] and eVITTA [12] are often employed to identify secondary metabolites. MetOrigin further advances this field by integrating metagenome and metabolome data, streamlining the identification of microbial metabolites and their corresponding bacterial sources. This system enhances both the efficiency and accuracy of microbial biomarker discovery.

As research in the fields of metagenomics and metabolomics continues to advance, there is a growing demand for more sophisticated analytical tools to interrogate the complex interactions between microbiome and their metabolites. In response to user feedback and emerging needs in this field, MetOrigin 2.0 has been developed with a substantial upgrade and significant improvement over the original MetOrigin. This new version includes the following key enhancements:

(1) Database Upgrade: The MetOrigin database has been updated with the latest data from seven public metabolite databases.
(2) Quick Search Module: A new module allows users to quickly identify bacteria associated with a metabolite of interest.
(3) Expanded Metabolite Origins: The classification of metabolite origins has been refined and expanded into five main categories: host, microbiota, food, drug, and environment.
(4) Orthology Analysis Module: This new feature connects metabolic enzyme genes with their associated bacteria.
(5) Mediation Analysis Module: A new module to explore potential causal relationships among bacteria, metabolites, and phenotypes.

All these enhancements are contained in MetOrigin 2.0, which is freely available at http://metorigin.met-bioinformatics.cn/. In addition, key features in existing modules have been significantly enhanced by providing customized options and upgrading the graphical output. We have also updated tutorials and frequently asked questions (FAQs) to the website to address common user questions and facilitate easier interaction with the platform.

## RESULTS

### Overview of MetOrigin 2.0

**FIGURE 1:**
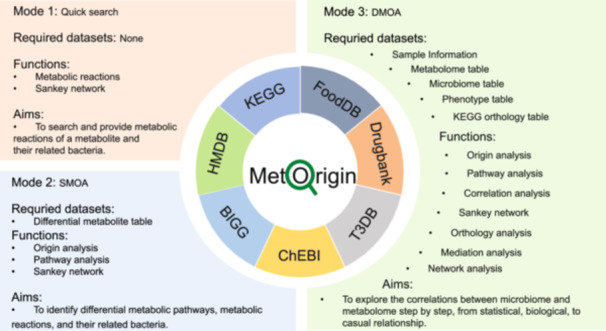
The main features of MetOrigin 2.0. BIGG, Biochemical Genetic and Genomic Knowledgebase; ChEBI, Chemical Entities of Biological Interest; Drugbank, Drugbank Knowledgebase; DMOA, Deep MetOrigin Analysis; F oodDB, Food Database; HMDB, Human Metabolome Database; KEGG, Kyoto Encyclopedia of Genes and Genomes; SMOA, Simple MetOrigin Analysis; T3DB, Toxin and Toxin Target Database.

MetOrigin 2.0 is an advanced, web‐based pipeline designed to facilitate step‐by‐step data integration and analysis for microbiome and metabolome studies. The platform's core functionalities are divided into three primary modes, each tailored to specific research needs based on the availability of microbiome and metabolome datasets. The curated MetOrigin database in the backend powers all three modes of data analysis. These modes are illustrated in Figure 1 and summarized as follows.

(1) Quick Search: This new mode enables researchers to efficiently determine the relationship between specific compounds/metabolites and bacteria without the need to upload any data. By querying the MetOrigin database, users can quickly obtain a list of metabolic reactions and their associated bacteria.
(2) Simple MetOrigin Analysis (SMOA): SMOA is designed for users who have a table of differential metabolites from a metabolomics study. Consistent with the previous version, this mode offers origin analysis to trace the sources of metabolites, pathway analysis to map metabolic processes, and Sankey network visualization to explore the microbiota linked to each metabolic reaction.
(3) Deep MetOrigin Analysis (DMOA): DMOA is the most comprehensive mode, comprising seven distinct modules. It requires three initial input files: a “Sample Info” table with sample and grouping data, a “Metabolite” table with compound details, and a “Microbiome” table with microbial annotations and abundance data from sequencing analysis. In addition to the features provided in the first version (origin analysis, pathway analysis, correlation analysis, Sankey network, and network analysis), DMOA introduces two new features. The first is orthology analysis, which connects metabolic enzyme genes with bacteria, requiring a KEGG orthology information table from metagenomics. The second is mediation analysis, which explores potential causal relationships among bacteria, metabolites, and phenotypes, requiring a phenotype table.

The key functions in MetOrigin 1.0 and 2.0 have been listed and compared in Table S1. Before conducting SMOA and DMOA, the platform offers data pretreatment options, including missing value imputation, scaling, and normalization. MetOrigin 2.0 is hosted on a cloud server and developed as an R Shiny application. All the statistical analyses and visualizations are performed in R (Version 3.6.0). A summary of R functions and packages has been provided in Table S2.

### MetOrigin database

MetOrigin integrates data from seven well‐known databases that are rich in metabolite sources and organism information, including the Kyoto Encyclopedia of Genes and Genomes (KEGG) [13], Human Metabolome Database (HMDB) [14], Biochemical Genetic and Genomic Knowledgebase (BIGG) [15], Chemical Entities of Biological Interest (ChEBI) [16], Food Database (FoodDB) [17], Drugbank Knowledgebase (Drugbank) [18], and Toxin and Toxin Target Database (T3DB) [19]. Since its introduction, MetOrigin has continuously updated its database, actively incorporating updates from public resources to expand the scope of metabolite information.

**FIGURE 2:**
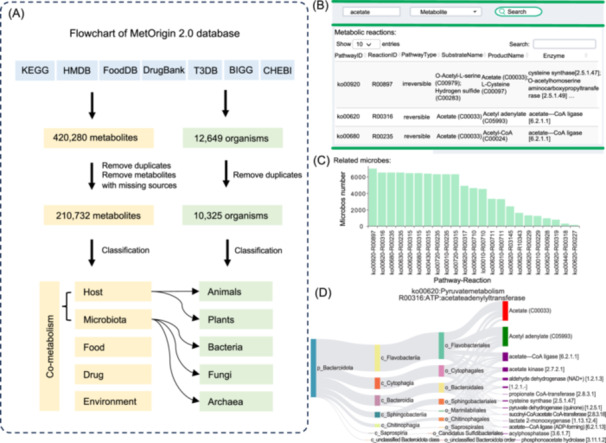
MetOrigin 2.0 database flowchart and main features of quick search. (A) Flowchart of MetOrigin 2.0 database. (B) Screen shot of quick search for “acetate” and a table of its metabolic reactions. (C) Bar plot showing the total number of related bacteria for each metabolic reaction of metabolite acetate. (D) Sankey network for the ko00620‐R00316 pathway. ATP, adenosine triphosphate; BIGG, Biochemical Genetic and Genomic Knowledgebase; ChEBI, Chemical Entities of Biological Interest; Drugbank, Drugbank Knowledgebase; FoodDB, Food Database; HMDB, Human Metabolome Database; KEGG, Kyoto Encyclopedia of Genes and Genomes; T3DB, Toxin and Toxin Target Database.

The comprehensive procedure for collecting, cleaning, and processing raw data on metabolites and organisms from these seven databases is illustrated in Figure 2A. First, a total of 420,280 metabolites were collected, of which 210,732 are “nonredundant” metabolites linked to at least one database and have specific source information that can be categorized into five groups: host (mammals), microbiota (archaea, fungi, bacteria), food (food & plants), drug, and environment (toxins & pollutants). Notably, a single metabolite may belong to multiple categories. For instance, metabolites classified under both host and microbiota categories are identified as “host‐microbiota” co‐metabolites. Second, a total of 12,649 different organisms for host and microbiota were collected, of which 10,325 are “nonredundant” organisms at species that are further classified into animals, plants, bacteria, fungi, and archaea. Notably, a total of 3211 microbial strains have been added in this version, facilitating the origin analysis and Sankey network visualization. Overall, MetOrigin 2.0 has expanded significantly, incorporating 19,701 additional metabolites and 3254 more organisms compared to the first version. Detailed updates on the number of metabolites and organisms from seven databases compared to the first version are summarized in Tables S3 and S4.

### Quick search

The Quick Search feature in MetOrigin 2.0 enables users to perform a quick search of any compound or metabolite present in the database, rapidly gaining insights into its related metabolic reactions, corresponding metabolic enzymes, and the bacteria that participate in these reactions. As multiple metabolic reactions can be associated with a single metabolite, the platform determines the degree of bacterial involvement in these reactions through calculating the number of related bacteria present in the database. For any given metabolic reaction, MetOrigin 2.0 employs a Sankey network to provide a clear and comprehensive visualization of the connections between the queried metabolite and bacteria at their different taxonomic levels. The interactive feature of the Sankey plot can be further customized based on the user's preferences. For example, when searching for acetate, a well‐known microbial metabolite, the Quick Search module identifies a total of 23 metabolic reactions from eight different metabolic pathways (Figure 2B). The total number of related microbes ranged from 59 to 5083 for these metabolic reactions (Figure 2C). For each specific metabolic reaction, the Sankey network is automatically produced, showing related bacteria at different taxonomic levels and offering an intuitive and customizable visualization of these relationships (Figure 2D).

### Orthology analysis

**FIGURE 3:**
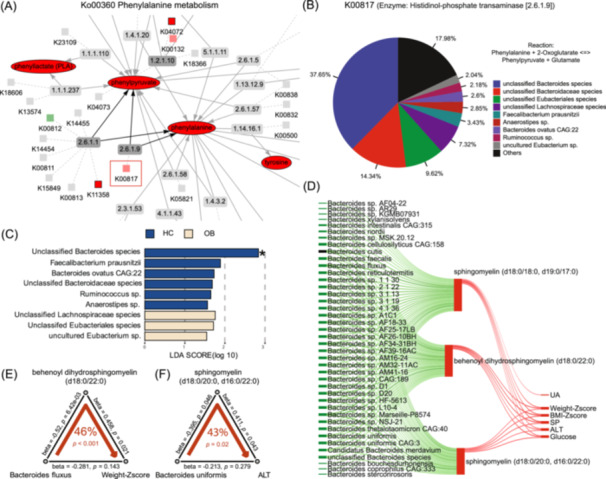
Orthology and mediation analyses of the gut microbiome and metabolome in a pediatric obesity study. (A) Metabolic network of phenylalanine metabolism. (B) Pie plot showing relative compositions of bacteria for metabolic enzyme gene K00817. (C) Bar plot of linear discriminant analysis (LDA) score from LEfse analysis. (D) Sankey network of correlations among *Bacteroides species*, sphingomyelins, and phenotypes. (E) Mediatory effect of behenoyl dihydrosphingomyelin on the relationship between *Bacteroides fluxus* and weight Z‐score. (F) Mediatory effect of sphingomyelin on the relationship between *Bacteroides uniformis* and ALT levels. ALT, alanine aminotransferase; BMI, body mass index; HC, healthy controls; OB, obesity; SP, systolic blood pressure; UA, uric acid.

Orthology Analysis is a new module introduced in MetOrigin 2.0, which provides a deeper understanding of unique metabolic characteristics and microbial enzyme‐metabolite interactions. This feature connects metabolites with KEGG Orthology (KO) markers based on the metabolic reactions in the database [20, 21]. First, significant metabolic pathways are initially identified through metabolic pathway enrichment analysis. Then, MetOrigin 2.0 constructs a network for each specific metabolic pathway by integrating available KO information. This network connects metabolites as substrates and products, reactions (enzymes), and their related KO genes (Figure 3A,B). Differential metabolites and their microbial enzyme genes are highlighted within the network when their differences between two groups reach the statistical significance. This module also allows for the identification of bacteria with enzyme genes (KO genes) responsible for specific metabolic reactions or processes. The relative compositions of bacteria to an enzyme gene are assessed based on their expression levels, which are further compared using discriminant analysis (Figure 3C,D).

### Mediation analysis

Mediation Analysis is a powerful tool for exploring the causal relationships between independent and dependent variables through mediators. This approach has been increasingly used in clinical research, particularly for identifying risk factors or early indicators of critical diseases [22, 23, 24]. In MetOrigin 2.0, mediation analysis is employed to unravel the complex connections between GM alterations and disease occurrence, with metabolites acting as mediators. This analysis enhances our understanding of the adaptive and feedback mechanisms of the microbiota in response to host pathogenetic factors. The mediation analysis module begins with Spearman correlation analysis on microbiome, metabolome data, and phenotype parameters (Figure S1A,B). Metabolites that correlate with both microbiome and phenotype parameters are identified and visualized in a Sankey network (Figure S1C). Guided by the Sankey network, users can subsequently select a specific metabolite of interest for further validation through mediation analysis, revealing functional insights and potential causality regarding the role of the gut microbiome in human health or disease onset (Figure S1D).

### Case study and performance validation

Next, we analyzed the gut microbiome and metabolome on a pediatric study of obesity with metabolic dysfunction‐associated steatotic liver disease (MASLD) that had been reported in the original MetOrigin, aiming to present the workflow and highlight the new features of MetOrigin 2.0 [1]. First, a total of 923 identified metabolites were initially classified into five groups: 240 host (human) related metabolites, 351 microbial metabolites, 217 drug‐related metabolites, 642 food‐related metabolites, and 51 environment‐related metabolites (Figure S2A). Among which, 217 metabolites are host‐microbiota co‐metabolites (Figure S2B). Second, the metabolic pathway enrichment analysis showed that there were three significant metabolic pathways for host metabolites, two for microbial metabolites, and 31 for host‐microbiota co‐metabolites by comparing the obesity group with healthy controls (Figure S2C,D).

The phenylalanine metabolism (Ko00360) was the most significant metabolic pathway for microbial metabolites, which was further explored to illustrate the new features of MetOrigin 2.0. In the orthology analysis, a network of phenylalanine metabolism was produced by connecting differential metabolites and KO genes (*p* < 0.05). The identified metabolites and KO genes without statistical significance were also mapped for reference (Figure 3A). Users can zoom in each part of the network. K00817 corresponds to the metabolic reaction enzyme histidinol‐phosphate transaminase [EC: 2.6.1.9] that participates in the metabolic reaction of phenylpyruvate and phenylalanine. The pie plot of different bacteria for K00817 based on their relative compositions showed that the top three dominant ones were *Bacteroides species, Bubacteriales species, and Lachnospiraceae species* (Figure 3B). Of which, the relative abundances of *Bacteroides species* were significantly expressed between two groups indicated by the discriminant analysis (Figure 3C). In the mediation analysis, six differential metabolites were identified due to their significant correlations with both microbiome and phenotype in the Sankey network (Figure S1C). Subsequently, we identified that *Clostridium species* may contribute to the development of obesity by increasing the N‐acetylpheylalanine level (30%, *p* < 0.001) (Figure S1D).

To further validate the findings generated by MetOrigin, we focused on sphingolipids, well‐known host‐microbiota metabolites, for targeted validation. Sphingolipids are crucial signaling molecules in mammals, playing key roles in the development of metabolic disorders [25]. Previous studies have demonstrated that sphingolipids can also be produced by *Bacteroidetes*, which influence host hepatic metabolism [26]. In the case study, we identified that a number of sphingomyelins in the sphingolipid catabolism were consistently increased in subjects with MASLD, which were classified as metabolites from host‐microbiota co‐metabolism by MetOrigin. The Sankey network analysis further revealed that sphingomyelins were positively correlated with the clinical liver injury biomarker alanine aminotransferase (ALT), as well as other metabolic markers (Figure 3D). Notably, *Bacteroides species* were significantly reduced in MASLD subjects and exhibited negative correlations with these sphingomyelins, suggesting an imbalance of sphingolipid biosynthesis and catabolism pathways [27]. Subsequently, the mediation analysis indicated that *Bacteroides fluxus* may contribute the weight loss by affecting the serum levels of behenoyl dihydrosphingomyelin (d18:0/22:0) (46%, *p* < 0.001); *Bacteroides uniformis* may play a protective role in alleviating liver damage by decreasing the level of sphingomyelin (d18:0/20:0, d16:0/22:0) (43%, *p* = 0.02) (Figure 3E,F). These results strongly support the protective effects of *Bacteroidetes species* in MASLD through modulating the homeostasis of host‐microbiota sphingolipid metabolism.

## DISCUSSION

Since its initial release, MetOrigin has been successfully applied across many research fields, enabling researchers to identify potential microbial metabolite biomarkers. In a clinical study, metagenomics and plasma metabolomics analyses were performed on mouse models to evaluate the effect of human fecal microbiota transplantation (FMT) on autistic symptoms [28]. MetOrigin analysis indicated the plasma compound pyridoxal, a natural form of vitamin B6, was strongly associated with multiple *Bacteroides species*, as shown through statistical and biological Sankey networks. This finding suggests that *Bacteroides species* play a key role in vitamin B6 metabolism in the FMT group. Further supplementation of vitamin B6 improved social behaviors in the mouse models, highlighting a potential mechanism for FMT's therapeutic role in autism spectrum disorder. In animal science, MetOrigin was used to reveal that *Selenomoas ruminantium* exhibited the most number of differential metabolic functions, potentially influencing feed utilization efficiency of dairy cows, which was also cross‐validated by metaproteomics analysis [29]. These findings were also cross‐validated by metaproteomics analysis, further strengthening the role of *Selenomonas* in improving livestock productivity. In a nutrition intervention study, MetOrigin helped identify that a black rice diet could alleviate colorectal cancer progression. The analysis showed an augmentation of probiotic strains, including *Bacteroides uniformis* and *Lactobacillus*, along with their microbial metabolites involved in the tryptophan metabolism pathway [30]. In environmental research, MetOrigin was applied to study the uptake and distribution of polystyrene nanoplastics (PS‐NPs) in zebrafish larvae, focusing on their toxic effects on the intestine [31]. The analysis uncovered significant correlations between bacterial flora, such as *Pedobacter* and *Bacillus*, and metabolites involved in glycolysis/gluconeogenesis pathways. The disruption of glycolipid metabolism after PS‐NP exposure was further validated through transcriptomics and proteomics, suggesting that polystyrene nanoplastics impair intestinal health, disturb microbiota, and induce metabolic disorders. Collectively, these studies demonstrate the power of MetOrigin in biomarker discovery, with subsequent experiments consistently validating the reliability of the findings produced by its pipeline.

The new version of MetOrigin aims to further advance the biomarker discovery of microbial metabolites and their related bacteria. One of the most notable features of the latest version is its flexibility and versatility, which is achieved through offering three distinct modes of operation: Quick Search, SMOA, and DMOA. These modes empower users to perform analyses at any stage of their research from preliminary exploration to in‐depth interrogation. Quick Search mode enables users to perform a comprehensive “survey” on metabolites, metabolic reactions, and associated bacteria before commencing omics studies. This mode is especially beneficial in expediting the preliminary exploration phase, providing a solid foundation for subsequent investigations, and serving as a complementary tool to the literature review process. Following the completion of a metabolomics study, the SMOA mode allows users to distinguish the origins of differential metabolites, conduct pathway analyses specific to microbial metabolites, and identify connections between bacteria and each metabolic reaction. This mode leverages a more focused analysis to refine the research scope using real‐world data set, therefore enhancing the relevance and applicability of the findings. This approach effectively guides researchers toward more informed and impactful discoveries in their field. When both microbiome and metabolome studies have been conducted, the DMOA mode offers a comprehensive suite of seven functions to explore the intricate relationships between microbiome and metabolome data at various levels and dimensions. The ideal of this mode is to unravel the causal relationships through extensive and in‐depth analysis, ultimately contributing to a more profound understanding of the underlying mechanisms that influence human health.

**FIGURE 4:**
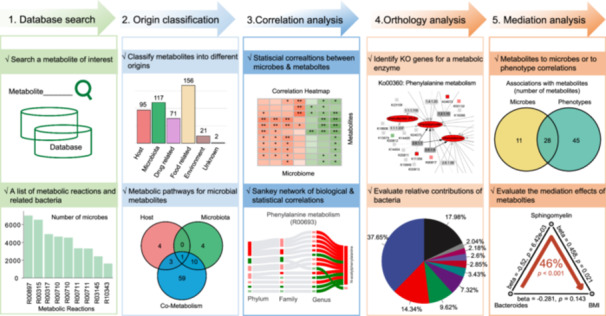
Summary of step‐by‐step strategy for exploring microbiome‐metabolome interactions in MetOrigin 2.0. BMI, body mass index; KO, KEGG Orthology.

MetOrigin 2.0 stands out for its systematic, step‐by‐step strategy for exploring microbiome‐metabolome interactions, significantly enhancing the efficiency of biomarker discovery and validation processes. The integration analysis in MetOrigin 2.0 operates at five distinct levels (Figure 4): (1) Database search: bacteria of a specific metabolite and its metabolic reaction are identified based on comprehensive database searches; (2) Metabolite Classification: metabolites are categorized by origins, allowing for further differential and pathway analysis; (3) Correlation Analysis: both biological and statistical correlations between microbiome and metabolome data are evaluated and integrated into a Sankey network, yielding a visual representation of these findings; (4) Orthology Analysis: specific bacteria involved in metabolic processes are pinpointed by connecting microbial enzymes, gaining additional understanding of these complex relationships; (5) Mediation analysis: the causal role of the microbiome in contributing to a phenotype through metabolite mediation is deciphered at this level, providing valuable insights into the underlying mechanisms. Collectively, these five primary levels of analysis offered by MetOrigin 2.0 empower researchers to systematically explore and understand the intricate relationships between microbiome and metabolome data.

Considering thousands of unknown secondary metabolites in the microbiome, current bioinformatics approaches and tools for predicting these secondary metabolites span various fields, including genomics, proteomics, transcriptomics, and metabolomics data [10]. Each tool has its distinct strengths and limitations in terms of scalability and underlying principles. As shown in Table 1, the key features of MetOrigin 2.0 are compared with other notable web‐based tools [11, 32, 33, 34]. Future advancements in microbial metabolite prediction will hinge on at least three critical factors: omics data integration, database robustness, and visualization capabilities. First, while many existing tools focus on a single omics domain, the integration of multi‐omics data offers a more comprehensive and enriched view of microbial interactions. Secondly, database development plays a crucial role in supporting data integration and metabolite prediction. Third, the ability to visualize microbial metabolite interactions is crucial for gaining deeper insights. MetOrigin has effectively captured these key features by integrating metagenomic and metabolomic data, connecting the curated backend database, and providing innovative visualization features, including Sankey networks, KO‐metabolite pathways, and microbe‐metabolite networks. To summarize, microbial metabolite prediction is a growing field that benefits from advancements in omics data integration, database infrastructure, and visualization techniques.

**TABLE 1:** Comparison of key features of MetOrigin 2.0 with other representative web‐based tools for metabolite prediction.

Feature	MetOrigin 2.0	antiSMASH	MelonnPan	OmicsNet	Amon
Focus area	Origin analysis of microbial metabolites via metagenome and metabolome information	Prediction of secondary metabolite biosynthetic gene clusters	Prediction of metabolic potential in microbial communities	Multi‐omics network analysis	Origin analysis of metabolites via genomic information
Key features	Integrates multi‐omics data for origin analysis	Comprehensive biosynthetic pathway predictions	Machine learning‐based predictive accuracy	Extensive network‐based analysis	Integrates multi‐omics data for origin analysis
Data integration	Microbiome and metabolome data	Primarily genomic data	Genomics and metabolomics	Genomics, transcriptomics, proteomics, metabolomics	Microbiome and metabolome data
Database search	Provides metabolite origin prediction	Link to independent antiSMASH database	/	/	/
Visualization	Advanced visualizations (e.g., Sankey network)	Basic gene cluster visualization	Limited visualizations	Extensive network visualizations	Limited metabolic pathway visualization
Predictive accuracy	Strong for origin analysis	High for biosynthetic clusters	High for metabolic potential	Moderate, focused on network correlations	Strong for origin analysis
Scale of application	Focused on gut microbiome and metabolome studies	Broad, covering various organisms and biosynthetic pathways	Broad, applicable to various microbiomes	Broad, applicable to various omics layers	Focused on gut microbiome and metabolome studies
Network analysis	Extensive	/	/	Extensive	Limited
Pathway analysis	Provides metabolic pathway	/	/	/	Provides metabolic pathway
Origin analysis	Host and microbiota origin analysis	/	/	/	Host and microbiota origin analysis
Software type	Web‐server, software	Web‐server, software	Software	Web‐server, software	Python package
User‐friendly interface	Specialized, with detailed analysis outputs	User‐friendly for gene cluster identification	User‐friendly, with machine learning integration	Complex requires expertize in network biology	/

Finally, it is important to recognize that MetOrigin 2.0 keeps evolving, with potential for future enhancements in its features and functions. In future updates, we aim to enrich the database with more comprehensive host information, supporting the diverse needs of researchers from various backgrounds. Additionally, we will continuously update and expand the sources of metabolites by incorporating data from literature through text mining and natural language processing. Recent advancements in culturomics have led to the isolation of numerous microbial colonies [35]. Moreover, metabolic profiling of bacterial strains using mass spectrometry‐based metabolomics has enabled the identification and quantification of their microbial products [36]. Such breakthroughs have discovered novel bacteria, metabolites, and metabolic processes, providing valuable insights into microbial metabolites and their origins. Thus, this wealth of accurate data on bacterial metabolic activities will be integrated into MetOrigin, facilitating users to validate their findings more effectively.

## AUTHOR CONTRIBUTIONS

**Gang Yu**: Software; methodology; data curation; investigation; visualization; writing—original draft; resources. **Cuifang Xu**: Software; methodology; data curation; investigation; visualization; formal analysis. **Xiaoyan Wang**: Data curation; validation; visualization. **Feng Ju**: Validation; writing—review and editing; formal analysis. **junfen fu**: writing—review and editing; project administration; supervision. **Yan Ni**: Conceptualization; methodology; software; validation; investigation; writing—original draft; writing—review and editing; funding acquisition; supervision; project administration; formal analysis; resources; visualization.

## CONFLICT OF INTEREST STATEMENT

The authors declare no conflict of interest.

## ETHICS STATEMENT

The ethics application (No. 2021‐IRB‐038) was approved by the Medical Ethics Committee of Children's Hospital Affiliated to Zhejiang University School of Medicine, which was also registered in the Chinese Clinical Trial Registry (ChiCTR2100045616).

## Supporting information


**Figure S1:** Mediation analysis workflow.
**Figure S2:** Origin analysis and pathway enrichment analysis (MPEA) of case study.

**Table S1:** Overview of the main functions of MetOrigin in version 1.0 and 2.0.
**Table S2:** List of R packages and functions applied for each step of MetOrigin analysis.
**Table S3:** Comparison of metabolite information in MetOrigin database 1.0 and 2.0.
**Table S4:** Comparison of host and microbiota information in MetOrigin database 1.0 and 2.0.

## Data Availability

All data should have been uploaded to GSA (https://ngdc.cncb.ac.cn/gsa/). All the sequencing data have been deposited in GSA under accession number CRA013297 (metagenome sequencing), BioProject accession number PRJCA020992 (https://ngdc.cncb.ac.cn/bioproject/browse/PRJCA020992). The data and scripts used are saved in GitHub (https://github.com/yanni617/MetOrigin_V2). Supplementary materials (figures, tables, scripts, graphical abstract, slides, videos, Chinese translated version, and update materials) may be found in the online DOI or iMeta Science http://www.imeta.science.
