# Supplementary material for: MetOrigin 2.0: Advancing the discovery of microbial metabolites and their origins
Source: Imeta. 2024 Nov 6;3(6):e246. doi: 10.1002/imt2.246 (PMC11683456; doi:10.1002/imt2.246)
Supplement: Supplementary file 1 — Figure S1: Mediation analysis workflow. Figure S2: Origin analysis and pathway enrichment analysis (MPEA) of case study. [file IMT2-3-e246-s002.docx]

**Supporting information to**

**MetOrigin 2.0: Advancing the discovery of microbial metabolites and their origins**

**Running title**: MetOrigin 2.0 explores microbial metabolites and their origins

Gang Yu^1^, Cuifang Xu^1^, Xiaoyan Wang^1^, Feng Ju^2^, Junfen Fu^1*^, Yan Ni^1*^

^1^Children’s Hospital, Zhejiang University School of Medicine, National Clinical Research Center for Child Health, Hangzhou 310052, China.

^2^Key Laboratory of Coastal Environment and Resources of Zhejiang Province, School of Engineering, Westlake University, Hangzhou 310024, China

***Correspondence:** [yanni617@zju.edu.cn](mailto:yanni617@zju.edu.cn) (Yan Ni), fjf68@zju.edu.cn (Junfen Fu)

**Supplementary figures**


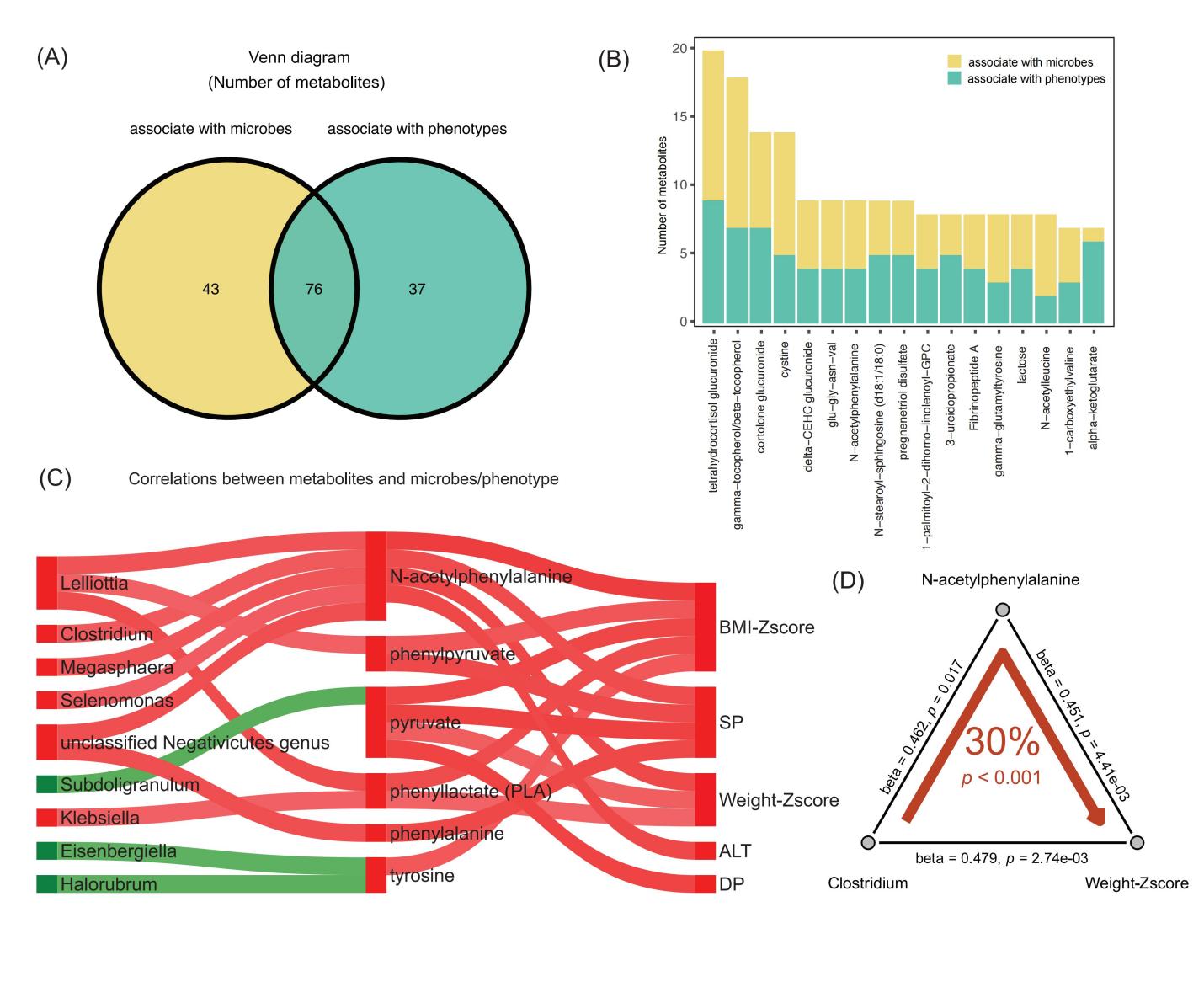


**Figure S1 Mediation analysis workflow.** (A) Venn diagram of differential metabolites that are significantly correlated with both microbes and phenotype parameters. (B) Bar plot showing the total number of correlated microbes or phenotype parameters for selected metabolites. (C) Sankey network of correlations among microbes, metabolites, and phenotypes. (D) Mediatory effect of N-acetylphenylalanine on the relationship between *Clostridium* and obesity. BMI, body mass index; SP, systolic blood pressure; ALT, alanine aminotransferase; DP, diastolic blood pressure.

**
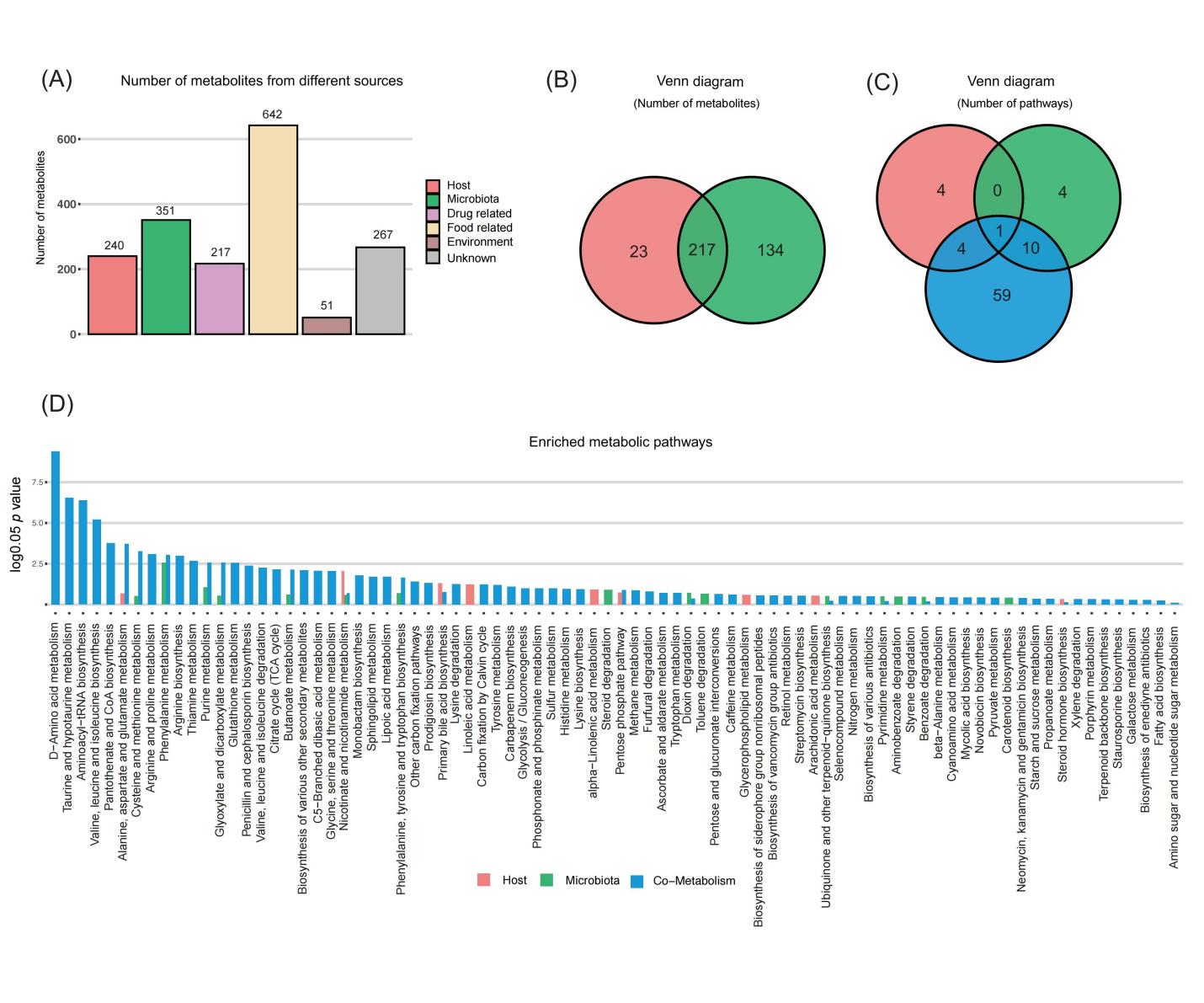
**

**Figure S2 Origin analysis and metabolic pathway enrichment analysis (MPEA) of case study.** (A) Bar plot showing the number of metabolites across different categories; (B) Venn diagram illustrating the number of metabolites from host, microbiota, and co-metabolism; (C) Venn diagram showing the number of enriched metabolic pathways from origin-based MPEA analysis; (D) MPEA analysis based on subgroup of metabolites from the host, microbiota, and co-metabolism.
